# Spatial Selectivity in Cochlear Implants: Effects of Asymmetric Waveforms and Development of a Single-Point Measure

**DOI:** 10.1007/s10162-017-0625-9

**Published:** 2017-07-28

**Authors:** Robert P. Carlyon, John M. Deeks, Jaime Undurraga, Olivier Macherey, Astrid van Wieringen

**Affiliations:** 1 MRC Cognition and Brain Sciences Unit, 15 Chaucer Rd, Cambridge, CB1 3DA UK; 2 ExpORL, Department of Neurosciences, KULeuven, Herestraat 49 bus 721, 3000 Leuven, Belgium; 3 LMA-CNRS, UPR 7051, Aix-Marseille University, Centrale Marseille, 4, Impasse Nikola Tesla, CS40006, 13453 Marseille Cedex 13, France

**Keywords:** cochlear implants, spatial selectivity

## Abstract

Three experiments studied the extent to which cochlear implant users’ spatial selectivity can be manipulated using asymmetric waveforms and tested an efficient method for comparing spatial selectivity produced by different stimuli. Experiment 1 measured forward-masked psychophysical tuning curves (PTCs) for a partial tripolar (pTP) probe. Maskers were presented on bipolar pairs separated by one unused electrode; waveforms were either symmetric biphasic (“SYM”) or pseudomonophasic with the short high-amplitude phase being either anodic (“PSA”) or cathodic (“PSC”) on the more apical electrode. For the SYM masker, several subjects showed PTCs consistent with a bimodal excitation pattern, with discrete excitation peaks on each electrode of the bipolar masker pair. Most subjects showed significant differences between the PSA and PSC maskers consistent with greater masking by the electrode where the high-amplitude phase was anodic, but the pattern differed markedly across subjects. Experiment 2 measured masked excitation patterns for a pTP probe and either a monopolar symmetric biphasic masker (“MP_SYM”) or pTP pseudomonophasic maskers where the short high-amplitude phase was either anodic (“TP_PSA”) or cathodic (“TP_PSC”) on the masker’s central electrode. Four of the five subjects showed significant differences between the masker types, but again the pattern varied markedly across subjects. Because the levels of the maskers were chosen to produce the same masking of a probe on the same channel as the masker, it was correctly predicted that maskers that produce broader masking patterns would sound louder. Experiment 3 exploited this finding by using a single-point measure of spread of excitation to reveal significantly better spatial selectivity for TP_PSA compared to TP_PSC maskers.

## **INTRODUCTION**

An important limitation on the ability of cochlear implant (CI) listeners to understand speech, particularly in noisy situations, is the poor spatial selectivity produced by contemporary electrode designs and stimulation methods. Ideally, each intra-cochlear electrode would excite a discrete set of auditory nerve fibres, each with a restricted range of characteristic frequencies (CFs) that varied monotonically with electrode position. However, the results of psychophysical and speech perception experiments have shown that the effective spread of excitation from each electrode is rather broad, such that the number of independent channels of information conveyed by the electrode array is considerably smaller than the number of physical electrodes (Chatterjee and Shannon 1998; Friesen et al. 2001; Kwon and van den Honert 2006). This reduced spatial selectivity impairs not only the identification of spectral features necessary for the identification of speech sounds but also the usefulness of cues such as onset differences that, for normal-hearing listeners, provide a powerful means for the perceptual separation of competing sounds (Carlyon et al. 2007).

**FIG. 1:**
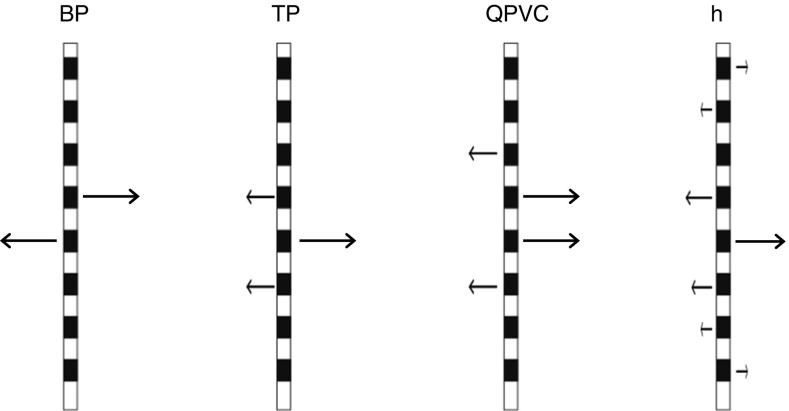
Schematic representation of bipolar (*BP*), tripolar (*TP*), quadrupolar virtual channel (*QPVC*), and all-polar (*AP*) modes of stimulation. The direction and length of each *arrow* indicates the polarity and amplitude of stimulation. Only eight electrodes are shown, for clarity.

Modern CIs stimulate each electrode in monopolar (MP) mode, in which current is returned via one or more extra-cochlear electrodes. Because MP stimulation produces a broad current spread within the cochlea, a number of more focussed methods have been developed. All of these methods involve returning all or part of the injected current via one or more intra-cochlear electrodes. Figure 1 illustrates four widely used methods, the first two of which are studied here: bipolar (BP), tripolar (TP), quadrupolar virtual channel (QPVC), and all-polar (AP). The figure illustrates the case where all of the current is returned by intra-cochlear electrodes; in practice, for TP and QPVC stimulation, a small proportion of the current is often returned by an extra-cochlear electrode, in which case the name is preceded by the word “partial” and abbreviated as pTP and pQPVC, respectively. As a number of authors have pointed out, the success of these new approaches has been, at best, mixed. One possible reason comes from the fact that every intra-cochlear electrode that is stimulated in a given configuration is likely to elicit neural excitation near that electrode. That is, whereas in MP mode the stimulation will arise from a single electrode, more focussed stimulation involves the stimulation of either two (BP), three (TP), four (QPVC), or all (AP) electrodes. Although one electrode is, for convenience, often described as the “active” electrode, the other “return” electrodes will be stimulated by a usually polarity-inverted and possibly scaled version of the waveform at the active electrode (Fig. 1). Activation by these electrodes may increase the width of the neural excitation pattern and may lead to those patterns containing two or more maxima. Strong evidence for multi-modal patterns of activation have come from in vitro and in vivo measurements of potential gradients, physiological tuning curves, and computational models (Kral et al. 1998; Litvak et al. 2007); perceptual evidence is discussed in the following sections.

A major goal of the present study was to determine whether the advantages of BP and TP stimulation could be retained whilst modifying the stimulation such that the neural activation pattern more closely resembles a single peak. To do so, we used asymmetric pulses and exploited the fact that CI listeners are sensitive primarily to anodic rather than to cathodic current (Macherey et al. 2008). In addition, our second and third experiments introduce a new method that may allow a fast comparison of the spread of excitation produced by different masker types, across the entire electrode array.

### Bipolar Mode

BP mode involves stimulating two electrodes with identical but polarity-inverted waveforms. The sensitivity of CI listeners to the symmetric pulses used clinically does not depend strongly on polarity, and so each electrode will stimulate the auditory nerve by an approximately equal amount. This can, in principle, lead to bimodal excitation patterns—an outcome that is made more likely by the fact that, at locations between the two stimulated electrodes, the current will cancel, thereby increasing the distance between the two maxima (Kral et al. 1998; Saoji and Litvak 2010; Macherey and Carlyon 2012). A recent simulation study with normal-hearing listeners has provided evidence that bimodal excitation patterns can impair speech perception (Mesnildrey and Macherey 2015).

Psychophysical evidence for bimodal excitation patterns has come from experiments measuring psychophysical tuning curves (“PTCs”; Nelson et al. 2008; Zhu et al. 2012), in which the levels of maskers centred on different electrodes are adjusted so as to mask a probe having a low level and presented to a fixed electrode. In contrast, masked excitation patterns—in which the masker is fixed and thresholds are measured as a function of probe position—are typically bimodal only when the separation between the two constituent electrodes is very wide (Lim et al. 1998; Chatterjee et al. 2006; Macherey et al. 2010). One reason for this may be that, at high levels, spread of excitation from the two electrodes in a closely spaced BP pair may cause them to blur together, leading to a unimodal pattern. It should also be noted that, near the peak of the masked excitation pattern, the signal level is also high; if this leads to the signal excitation pattern also being broad, this would hide any bimodality in the excitation pattern of the masker. Further evidence that bipolar stimuli presented at high levels produce bimodal excitation patterns only at wide separations comes from spread-of-excitation measures obtained using the electrically evoked compound action potential (“ECAP”; Undurraga et al. 2012).

**FIG. 2:**
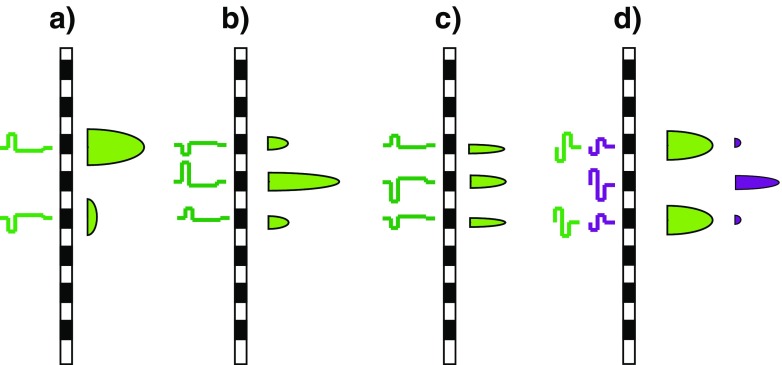
**a** How the bimodal excitation patterns that arise from BP stimulation with symmetric waveforms may be reduced by the use of pseudomonophasic waveforms. The waveforms are shown to the *left* of each stimulating electrode, and the schematic excitation patterns are shown to the *right* of the electrodes. **b**, **c** How the relative amplitudes of the central and side lobes of excitation, produced by tripolar stimulation, may be affected by the polarity of stimulation. **d** The situation in experiment 1 where the symmetric bipolar masker (*green*) is presented on electrodes (-1,1), thereby straddling the partial-tripolar probe (*purple*) on electrode 0.

Our proposed method for reducing the bimodality of the neural spread of excitation in BP mode is illustrated in Figure 2a, which shows the waveforms and schematic neural excitation patterns at the two electrodes of a BP pair, produced by an asymmetric pseudomonophasic pulse. It builds on the well-established finding that, above threshold, CI listeners are primarily sensitive to anodic current and that a pseudomonophasic pulse is more effective when the short high-amplitude portion is anodic than when it is cathodic (Macherey et al. 2006; Macherey et al. 2008; Macherey et al. 2010; Undurraga et al. 2010). In Figure 2a, this waveform is applied to the electrode shown towards the top of the plot, which would therefore be expected to elicit more excitation than the other electrode in the bipolar pair. The aim of experiment 1 was to determine whether bimodal excitation patterns could be observed by measuring PTCs for bipolar maskers for small (BP+1) electrode separations and whether this bimodality could be reduced by using asymmetric pulse shapes.

### Tripolar Mode

In TP mode, each of the two flanking electrodes is stimulated by a polarity-inverted and half-amplitude version of the waveform presented to the central electrode. Returning current via two flanking electrodes produces a more restricted current spread than in MP mode. However, a computational model proposed by Litvak et al. (2007) suggests that the neural spread of excitation may not always be reduced for TP compared to MP stimuli. According to the model, when neurons responding to the central electrode are saturated, the excitation pattern becomes increasingly dominated by the side lobes. As a result, for equally loud stimuli, the excitation pattern can become even *broader* with TP than with MP stimulation; this will depend on factors such as local neural survival, distance from the electrode to the surviving neurons, and overall stimulus level. Other computational models also predict that TP stimuli can, depending on these and other factors, produce trimodal neural excitation patterns (Goldwyn et al. 2010; Kalkman et al. 2015).

Our proposed method for manipulating the neural spread of excitation in TP mode is illustrated in Figure 2b, c. It uses pseudomonophasic pTP maskers in which the short high-amplitude phase presented to the central electrode is either anodic (“PS_A”, Fig. 2b) or cathodic (“PS_C”, Fig. 2c). The hypothesis is that the central lobe of excitation will be enhanced at the expense of the side lobes for the PS_A stimulus, with the opposite effect occurring for the PS_C masker. We measure masked excitation patterns for these two stimuli and for a comparison condition with a symmetric MP masker. In addition, the second part of the article introduces a fast method for determining which of two pulse trains produces the broader spread of excitation. The method is based on the assumption that, when two stimuli produce the same amount of masking for a probe presented on the masker electrode, the one with the broader excitation pattern will be louder because it produces more excitation elsewhere. This method has the additional advantage over masked excitation patterns and PTCs in that it allows an estimate of the spread of excitation evoked on all electrodes, including those at the ends of the array.

To summarise, our first two experiments manipulated masker polarity in an attempt to produce a more controlled spread of neural activation. Experiment 1 used bipolar maskers and measured psychophysical tuning curves for pTP signals. We chose pTP rather than MP signals in an attempt to limit the spread of excitation, on the assumption that, at the low sensation levels used for the probe, any side lobes would be below threshold. Because the excitation produced by the masker at the probe place is typically low when measuring PTCs, this method had the potential advantage of revealing bimodal patterns of excitation. This in turn made it possible to investigate the effect of masker polarity on each “lobe”. Experiment 2 measured masked excitation patterns for pTP maskers. One advantage of this approach is that the amount of masker excitation at the probe place can be quite high for masked excitation patterns. Polarity effects are generally larger at high excitation levels than close to threshold (Macherey et al. 2006; Macherey et al. 2008; van Wieringen et al. 2008). This may have increased our chances of seeing an effect of polarity on threshold, compared to the PTC procedure of experiment 1 where the masker excitation at the probe place would have been low, as the masker only had to mask a low-SL probe. A modified version of this method was then used in experiment 3 in a further test of the effect of stimulus polarity on the spatial spread of excitation in tripolar mode. The results of all three methods concurred in showing that polarity effects were on average small, but were usually significant and sometimes substantial for individual subjects. In addition, the results of the last two experiments validate the use of a novel time-efficient method for comparing the spatial selectivity of different stimuli.

## **EXPERIMENT 1: PTCs FOR BIPOLAR MASKERS**

### Method

Seven users of the HiRes 90K CI, manufactured by Advanced Bionics, took part: AB1, and AB102 to AB107. Throughout this article, subject numbers greater than 100 refer to patients implanted and tested in Belgium, whereas those lower than 100 were implanted and tested in Cambridge, England. Their details are given in Table 1. Stimuli were generated and controlled using the BEDCS software provided by Advanced Bionics, and by a customised version of the APEX software package that acted as a wrapper around the low-level BEDCS routines. All stimuli were checked using a test implant and digital storage oscilloscope.

**TABLE 1:** Details of the subjects who took part in the experiments. Subjects AB1 to AB6 were from Cambridge, UK (“Cam”) whilst subjects AB102 to AB106 were from Leuven, Belgium (“Leu”)

Subject	Centre	Age (years)	Electrode array
AB1	Cam	67	HiFocus1J
AB2	Cam	69	HiFocus1J
AB3	Cam	53	HiFocus1J
AB4	Cam	65	HiFocus1J
AB5	Cam	66	HiFocus1J
AB6	Cam	69	HiFocus ms
AB102	Leu	60	HiFocus 2
AB103	Leu	74	HiFocus Helix
AB104	Leu	64	HiFocus 2
AB105	Leu	62	HiFocus 2
AB106	Leu	62	HiFocus 2
AB107	Leu	66	HiFocus 2

PTCs were measured using a symmetric cathodic-1st pTP or TP probe; the proportion of current returned by the flanking electrodes depended on the amount of current needed for the probe to reach at least a “soft” level. It was 0.75 for listener AB1, 0.45 for listener AB104, and 1.0 for the other three listeners. Each probe pulse consisted of two 97-μs phases of equal amplitude and opposite polarity, with a zero inter-phase gap. The centre electrode was chosen to be near the middle of the array and was electrode 9 for subject AB105 and electrode 8 for all others. The probe had a total duration of 10 ms and consisted of three pulses at a rate of 344 pps. This low rate was chosen to be substantially lower than that used for the masker (see below) so as to avoid “confusion” effects that may occur when the masker and probe are presented to the same electrode and are otherwise identical, leading to the probe being mistaken for a continuation of the masker (Neff 1985; Cosentino et al. 2015).

Three different masker types were used, each of which consisted of a 200-ms 1031-pps pulse train presented in BP+1 mode (that is, with one unused electrode between the two electrodes that constituted each BP pair). The masker types differed only in their pulse shape and polarity, which is defined in terms of the waveform at the more apical electrode in each BP pair. They were as follows: (a) BP_SYM: a symmetric 97-μs/phase biphasic pulse, (b) BP_PSA: a pseudomonophasic pulse with an anodic short high-amplitude phase of 97 μs followed by a four times longer and one-quarter-amplitude cathodic phase, (c) BP_PSC: the same as for BP_PSA but with the opposite polarity, so that the short high-amplitude phase was cathodic on the more apical electrode. The position of the masker is described in terms of the position of its two constituent electrodes relative to the central electrode of the TP_SYM signal, designated as electrode 0. Five masker positions were tested: (-3,-1), (-2,0), (-1,1), (0,2), and (1,3). Note that the masker position (-1,1) straddles the probe position and might be expected to be ineffective if that masker produced a bimodal excitation pattern (Fig. 2d).

For the PTC measurements, each trial started with the masker at a low level. This level was increased after every two consecutive correct trials and decreased by the same amount after every incorrect trial. The change from an increasing to a decreasing level, or vice versa, was defined as a turnpoint. The level change was 12 μA for the first two turnpoints and was 4 μA for the remaining six turnpoints. Each trial continued until eight turnpoints had been completed, and the masker level at threshold (MLT) for that run was calculated from the mean of the last six turnpoints. MLTs were obtained for each electrode position and for each masker and then repeated in reverse order; this whole procedure was repeated again so that each final MLT was calculated from the mean of at least four runs. For subject AB1, who was tested first, two-interval forced-choice trials were used. We subsequently found that some subjects found this trial structure difficult, and so all other subjects were tested using a three-interval two-alternative forced-choice (3I2AFC) structure, in which the signal could appear in either the second or third intervals. The 3I2AFC structure is a type of “odd man out” trial, which allows the subject to identify the signal using any available perceptual difference. In all cases, the silent interval between trials was 700 ms.

For subject AB1, the probe was set to a level 3 dB above its detection threshold measured in quiet, estimated using three runs of a “two up one down” adaptive procedure (Levitt 1971). For the other subjects, we used a method designed to fix the probe at a level that could be just masked by the most distant maskers, without those maskers exceeding a comfortable loudness. To do this, we fixed maskers (-3,-1) and (1,3) at 90 % of their dynamic range, measured the masked threshold for the probe in the presence of each of these two maskers separately, and then selected the lowest of these two levels for the probe in the PTC measurements.

In addition, we obtained an estimate of sensitivity across the range of electrode positions used to measure the PTC. This was done by measuring the detection threshold in quiet for symmetric biphasic pulse trains having the same pulse rate (1031 pps) and duration (200 ms) as the masker, but presented in TP mode. We used TP rather than BP+1 stimuli in order to obtain more place-specific estimates of sensitivity; as argued in the “INTRODUCTION”, with BP stimuli it is not possible to know, a priori, whether the stimulus will be detected primarily near the apical or basal electrode of the pair. Detection thresholds were obtained using a 2IFC trial structure. Each run started with the signal well above the expected threshold, and its level was reduced following every two consecutive correct responses and increased after every incorrect response. The step size was 28 μA for the initial two turnpoints and 4 μA for the final six turnpoints. The procedure stopped after eight turnpoints, with the threshold for that run estimated from the last four turnpoints. Thresholds were obtained from the average of two runs for each electrode position.

### Results

**FIG. 3:**
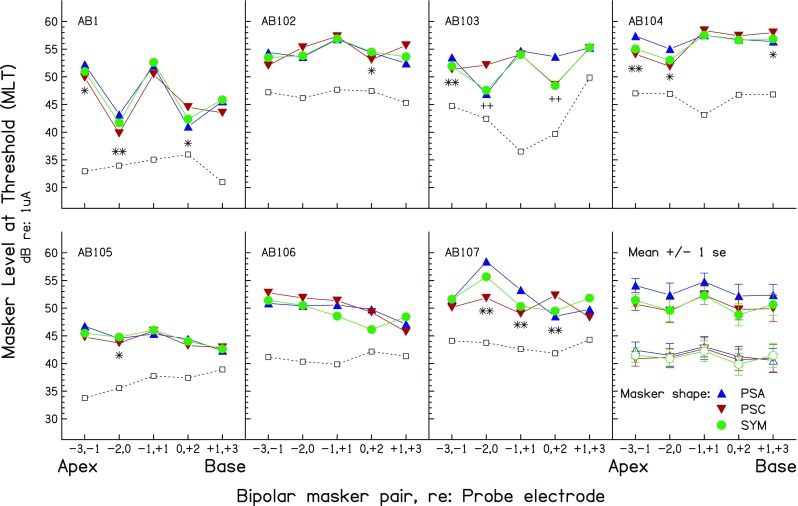
Forward-masked PTCs from experiment 1. The first seven panels show the results for individual subjects. Mean data are shown in the eighth (*bottom right*) panel for all subjects (*filled symbols*) or for all except subject AB1 (*open symbols*, shifted downwards by 10 dB for clarity). PTCs for the PSA, PSC, and SYM maskers are shown by the *upward triangles*, *downward triangles*, and *circles*, respectively. Points where the MLTs for the PSA and PSC maskers differed significantly at the 0.05 and 0.01 levels are shown by *single* and *double symbols*, respectively; *asterisks* indicate instances where the difference is in the predicted direction (PSA > PSC for apical maskers, PSA < PSC for basal maskers; see text) whereas *crosses* show differences in the opposite direction. The ordinate shows the MLT for the PTC measures. The values on the *ordinate* also show the detection threshold for each probe in quiet, shown by the *open squares* joined by *dashed lines*, which are shifted upwards by an arbitrary amount for each subject. These amounts are, in ascending order of subject number, −9, 0, −8, −9, −12, −3, and 0 dB.

MLTs for the three masker types are shown, as a function of masker position, for each subject in the first seven panels of Figure 3; average data are shown by the solid symbols in the final (bottom right) panel. It can be seen that the pattern of results differs across subjects, and a two-way (masker position X masker type) repeated-measures ANOVA revealed no statistically significant main effects or interactions (masker type: *F*(2,12) = 2.16, *p* = 0.18; masker position: *F*(4,24) = 2.10, *p* = 0.16; interaction: *F*(8,48) = 0.78, *p* = 0.52; throughout this article, the Huynh-Feldt sphericity correction is used and the uncorrected degrees of freedom are reported). There were also no main effects or interactions when the data from subject AB1, for whom a slightly different method was used, were excluded from the analysis (masker type: *F*(2,10) = 1.69, *p* = 0.23; masker position: *F*(4,20) = 0.98, *p* = 0.42; interaction: *F*(8,40) = 0.68, *p* = 0.58). Mean values with AB1’s data excluded are shown by the open symbols in the bottom right panel; they have been shifted downwards by 10 dB for clarity.

Despite the absence of significant effects when data from all subjects were combined, inspection of Figure 3 suggests the presence of substantial effects of masker type and position for individual subjects. We therefore analysed the data by performing separate univariate ANOVAs for the data of each subject, using the multiple runs for each condition as the error term. The results of these ANOVAs are shown in Table 2a and reveal that every subject showed a significant interaction between masker type and electrode position; this was true even for AB106, for whom the PTCs are flatter than for most other subjects. All subjects except AB106 also showed significant differences when only the data of the PSA and PSC conditions were analysed (Table 2b). Before discussing the results further, we consider two factors that could potentially influence the interpretation of the results.

**TABLE 2:** Part a) shows results of univariate ANOVAs on individual subjects’ results in all three conditions of experiment 1. This analysis is repeated for the PSA and PSC conditions only in part b). Part c) shows the results of univariate ANOVAs performed on each subject’s results in the SYM condition of experiment 1. The first column shows the main effect of electrode. The second two columns show the (uncorrected) significance levels of *t* tests between the MLTs for the masker on electrodes (-1,1) and, respectively, (-2,0) and (0,2). Comparisons that were significant with a *p* value of 0.001 or lower would have survived Bonferonni correction, whereas the two comparisons (both for subject AB2) significant at the *p* < 0.05 level would not have

a)			
Subject	Electrode effect	Masker effect	Interaction
AB 1^a^	*F*(4,45) = 109.9, *p* < 0.001	*F*(2,45) = 3.2, *p* = 0.05	*F*(8,45) = 3.5, *p* < 0.01
AB 102	*F*(4,60) = 11.3, *p* < 0.001	*F*(2,60) = 0.2, n.s.	*F*(8,60) = 2.8, *p* < 0.05
AB 103	*F*(4,45) = 234.4, *p* < 0.001	*F*(2,45) = 25.5, *p* < 0.001	*F*(8,45) = 43.4, *p* < 0.001
AB 104	*F*(4,60) = 99.9, *p* < 0.001	*F*(2,60) = 9.9, *p* < 0.001	*F*(8,60) = 14.6, *p* < 0.001
AB 105	*F*(4,60) = 35.0, *p* < 0.001	*F*(2,60) = 3.1, n.s.	*F*(8,60) = 2.3, *p* < 0.05
AB 106	*F*(4,60) = 39.2, *p* < 0.001	*F*(2,60) = 7.0, *p* < 0.01	*F*(8,60) = 6.7, *p* < 0.001
AB 107	*F*(4,69) = 37.4, *p* < 0.001	*F*(2,69) = 37.4, *p* < 0.001	*F*(8,69) = 10.6, *p* < 0.001
b)			
Subject	Electrode effect	Masker effect	Interaction
AB 1^a^	*F*(4,30) = 73.6, *p* < 0.001	*F*(1,30) = 5.4, *p* < 0.05	*F*(4,30) = 6.7, *p* = 0.001
AB 102	*F*(4,40) = 7.4, *p* < 0.001	*F*(1,40) = 0.4, n.s.	*F*(4,40) = 4.7, *p* = 0.003
AB 103	*F*(4,30) = 87.9, *p* < 0.001	*F*(1,30) = 6.2, *p* < 0.05	*F*(4,30) = 28.6, *p* < 0.001
AB 104	*F*(4,40) = 60.4, *p* < 0.001	*F*(1,40) = 11.4, *p* < 0.05	*F*(4,30) = 26.8, *p* < 0.001
AB 105	*F*(4,40) = 18.2, *p* < 0.001	*F*(1,40) = 3.9, n.s.	*F*(4,30) = 3.1, *p* < 0.05
AB 106	*F*(4,40) = 25.8, *p* < 0.001	*F*(1,40) = 1.8, n.s.	*F*(4,40) = 2.3, n.s.
AB 107	*F*(4,46) = 24.2, *p* < 0.001	*F*(1,46) = 24.1, *p* < 0.001	*F*(4,46) = 16.6, *p* < 0.001
c)			
Subject	Electrode effect	(-2,0) vs (-1,1)	(0,2) vs (-1,1)
AB 1^a^	*F*(4,15) = 98.6, *p* < 0.001	*p* < 0.001	*p* < 0.001
AB 102	*F*(4,20) = 4.4, *p* = 0.01	*p* < 0.05	*p* < 0.05
AB 103	*F*(4,15) = 403.6, *p* < 0.001	*p* < 0.001	*p* = 0.001
AB 104	*F*(4,20) = 41.6, *p* < 0.001	*p* < 0.001	n.s.
AB 105	*F*(4,20) = 32.3, *p* < 0.001	*p* = 0.001	*p* < 0.001
AB 106	*F*(4,20) = 42.3, *p* < 0.001	*p* < 0.001	*p* < 0.001
AB 107	*F*(4,23) = 18.6, *p* < 0.001	n.s.	n.s.

^a^A slightly different method was used for this subject

As shown in Figure 2, the spread of current from our symmetric TP probe consists of a narrow central lobe flanked by two smaller lobes of half the amplitude. We would expect, based on the fact that the probe was close to its detection threshold, that it would be detected from neurons close to the central lobe (Litvak et al. 2007), and there is indeed evidence that low-level TP probes produce narrower spreads of excitation than MP probes at the same sensation level (Bierer and Faulkner 2010). However, an exception might occur when the central probe electrode falls inside a dead region. The open squares connected by dashed lines in Figure 3 show the unmasked detection thresholds for TP signals as a function of electrode position; these have each been shifted vertically by an arbitrary amount, specified in the figure legend, in order to fit in each panel. It can be seen that, for all subjects, the probe falls either in a valley or in a flat region of the threshold profile. We therefore think it unlikely that the TP probe fell inside a dead region.

Another potential factor arises from the fact that, because the waveforms at each electrode of a BP stimulus are polarity-inverted versions of each other, they will tend to cancel each other at positions in between the two electrodes (Macherey et al. 2011; Saoji et al. 2013). As a result, the maxima in the current profiles may occur at positions that are slightly apical to the more apical electrode and slightly basal to the more basal electrode of the BP pair. Although this fact should be born in mind when interpreting PTCs obtained with BP maskers, we could not find any instances where it could explain anomalies in our data.

#### Symmetric Biphasic Masker

We analysed the results obtained with the symmetric biphasic masker in order to evaluate whether a bimodal pattern could be observed when the two electrodes in each bipolar pair were separated by only one unused electrode (cf. Nelson et al. 2008; Zhu et al. 2012). All subjects showed significant effects of electrode position for the SYM masker (Table 2c). Strong evidence for a bimodal pattern comes from the results of subjects AB1, AB102, and AB103, who showed markedly higher MLTs when the masker was on position (-1,1)—in which it “straddled” the probe central electrode (Fig. 2d), compared both to positions (-2,0) and (0,2), where one of the masker electrodes co-incided with the central electrode of the probe. This was also true, to a lesser extent, for subject AB105. Subject AB104 showed a higher MLT for masker (-1,1) than for masker (-2,0), but masker (0,2)—in which one electrode co-incided with the central probe electrode, also showed a high MLT. Subject AB107 appeared to show a different pattern of results, in which the lowest MLTs were for maskers at positions (-1,1) and (0,2). One reason why the predicted bimodal pattern may not occur for all subjects would be if the probe produced a fairly broad excitation pattern. For example, if that pattern extended from electrodes -1 to 1, then a BP masker presented on position (-1,1) would be effective and produce a correspondingly low MLT.

#### Pseudomonophasic Maskers

As noted above, analysis of the PSA and PSC data revealed that all subjects except AB106 showed a significant effect of masker type and of its interaction with electrode position (Table 2b). Our hypothesis was that, for maskers on the apical side of the probe, the MLT would be lower for the PSC than for the PSA masker type. This is because the more basal electrode in each pair would be the one closest to the probe and because, for the PSC masker, this electrode would be stimulated with the high-amplitude anodic phase (recall that masker polarity is defined relative to the more apical electrode in each pair). In contrast, the opposite effect was predicted for maskers basal to the probe. The asterisks and crosses in Figure 3 indicate Bonferonni-corrected pairwise comparisons where there was a statistically significant difference between the MLTs for the PSA and PSC maskers, with single and double symbols representing significance at the 5 and 1 % levels, respectively. (Throughout this article, we describe the results of the Bonferonni correction by multiplying the obtained probability values by the number of observations, rather than by dividing the criterion value by that number.) Of the 14 conditions where a significant difference was observed, 12 were in the predicted direction, as shown by the asterisks. However, two comparisons, both for subject AB103, were in the opposite direction (crosses).

Although the results of experiment 1 are consistent with previous evidence that most CI users are primarily sensitive to anodic current, there are two important qualifications to this conclusion. One of these, as noted above, comes from the data of subject AB103, which are more consistent with greater sensitivity to cathodic current. The other is that the effects, although often statistically significant, were generally quite small; where the data from the symmetric maskers were consistent with a bimodal excitation pattern, this was also generally true with the pseudomonophasic maskers. One possible reason for this, and for the anomalous effect of polarity for subject AB103, comes from the finding that, although markedly greater sensitivity to anodic current is consistently observed in loudness judgements at high overall loudness levels, unmasked thresholds are not markedly lower for PSA than for PSC stimuli (Macherey et al. 2006). Indeed, there is recent evidence that detection thresholds for some subjects can be significantly lower for cathodic than for anodic stimuli, suggesting that, at these low levels, some subjects may be more sensitive to cathodic current (Macherey et al. 2017). These findings are relevant to the interpretation of PTCs because, even when the maskers are quite loud, they produce, at their MLT, only enough excitation to mask a probe that is quite close to its detection threshold. For this reason, experiment 2 measured the masking patterns evoked by fixed high-level maskers, as a function of probe position.

## **EXPERIMENT 2: FORWARD-MASKED EXCITATION PATTERNS**

### Overview and Rationale

As noted above, there is evidence to suggest that the greater sensitivity to anodic than to cathodic current is larger and more consistent at high than at low excitation levels. Therefore, experiment 2 measured forward-masked excitation patterns, where the masker is fixed at a high level and probe threshold is measured as a function of probe position. This gave us the opportunity to measure masked thresholds on at least some probe electrodes—those fairly close to the masker—where the masker should produce a high level of excitation in the nearby neurons. The aim of the experiment was to determine whether one could produce a more restricted spread of excitation by using asymmetric maskers in TP mode. The rationale was to use a PS masker in which the waveform at the central electrode consisted of a PSA pulse and that at the flanking electrodes consisted of a PSC pulse. As illustrated in Figure 2b, this “TP_A” stimulus was expected to reduce the excitation at the flanking electrodes, compared to its polarity-inverted “TP_C” counterpart (Fig. 2c). (In fact, we used partial tripolar stimulation, but use the abbreviations “TP_A” and “TP_C” for brevity). Excitation patterns were measured for these two stimuli and for the stimulus used clinically in all contemporary CIs, namely a symmetric biphasic pulse presented in monopolar mode (“MP_SYM”). The probe was always presented in TP mode and had a symmetric biphasic shape, with the initial phase being anodic on the central electrode.

An important issue when comparing masked excitation patterns produced by different maskers arises when, as in the present case, setting the maskers to equal current is not expected to produce the same threshold when the probe is presented on the same electrode as the masker (the “on-site” probe). Several options are available to the experimenter. One is to present the maskers at the same current level and to scale the resulting forward-masked patterns so that they co-incide at their peaks (cf. McKay 2012). However, as Cosentino et al. (2015) have pointed out, this method assumes that Weber’s law holds at each place along the cochlea, so that the threshold for a probe on a given electrode can be taken as an accurate measure of the amount of excitation produced by the masker near that electrode. There is evidence to suggest that, for electric stimulation, this is not the case, with smaller changes in current being detectable at high overall excitation levels (e.g. Nelson et al. 1996). Alternatively, one could equate the maskers for equal loudness. However, this would not necessarily produce equal masking for the on-site probe, and so the scaling problem would remain. Furthermore, if one stimulus produces a more restricted spread of excitation, one may therefore end up increasing its current level to compensate for this reduced spread, thereby partially obscuring any difference between it and a stimulus that produces a broader spread. We therefore used a modified version of the method introduced by Macherey et al. (2010), in which the masker levels were initially adjusted so as to produce the same masked threshold for the on-site probe. The reasoning is that, as the resulting masked excitation patterns are aligned at their peaks, one can simply identify the more selective masker as the one that produces least masking at the other, “off-site”, electrodes. We also used this logic to test a simple method of comparing excitation pattern widths, based on the idea that, for maskers that produce the same amount of on-site masking, those that produce more off-site masking should sound louder.

A second issue arises from the fact that thresholds vary across the electrode array in the absence of the masker. We should stress that this does not affect our method for comparing the *relative* amounts of off-site masking for the different maskers; it is still the case that the more selective masker will produce less off-site masking than the others because the probe remains the same in all conditions. Instead of showing the probe threshold shifts produced by the maskers, we have chosen to plot the untransformed masked thresholds, with the unmasked thresholds provided on the same plots. This gives the reader access to the raw data, to the variation in absolute sensitivity across the electrode array, and to the total amount of masking (as the difference between the masked and unmasked thresholds).

### Method

Five users of the Advanced Bionics HiRes 90K implant (Table 1, AB1, AB2, AB3, AB4, and AB5) took part. Subject AB1 had participated in experiment 1. Subjects AB1, AB2, AB3, and AB4 took part in the study by Cosentino et al. (2015) and are described using the same abbreviations as in that study. Subject AB4 had normal hearing in her unimplanted ear and was subject C2 in the study by Carlyon et al. (2011).

All maskers consisted of 200-ms 1031-pps pulse trains. The current applied to the central electrode of each pulse in the TP_PSA masker (Fig. 2b) consisted of a 97-μs anodic phase immediately followed by a 384-μs one-quarter-amplitude cathodic phase. This masker was presented in partial tripolar mode, with 25 % of the current returned via an extra-cochlear electrode; as noted above, it is abbreviated as TP_PSA (rather than pTP_PSA) for brevity. The TP_PSC masker (Fig. 2c) was a polarity-inverted version of the TP_PSA masker. Each pulse of the MP_SYM masker consisted of a 97-μs/phase anodic phase followed immediately by a cathodic phase of the same amplitude and duration.

In the signal interval of each 2IFC trial, the masker was followed, after a 10-ms silent gap, by a 20-ms 200-pps anodic-leading symmetric biphasic probe. The probe was presented in partial tripolar mode with 25 % of the current returned via the case, which serves as an extra-cochlear electrode. The position of the central electrode of the probe was -2, 1, 0, 1, or 2 electrodes relative to that of the masker. Thresholds were also measured for these probes in the absence of any masker.

The masker was presented on electrode 8 for listeners AB1, AB3, and AB5. For the other two subjects (AB2 and AB4), electrode 4 was chosen, because electrode 8 was not included (locally disabled) in the program map for AB4, and generally higher thresholds for more basal electrodes led to audibility issues for AB5. The method of equating the masker levels so as to produce equal masking of the on-site probe differed slightly between subjects. In all cases, the procedure started by adjusting each masker so it was audible and then so that its loudness was at point 6 (“most comfortable”) of an 11-point scale. These levels are referred to as threshold and most comfortable level (MCL). For subjects AB1 and AB4, the method then proceeded as follows: (i) Fix the maskers at MCL and measure the masked threshold for the on-site probe for each of these maskers; (ii) take the lowest of these three masked thresholds, fix the on-site probe at that level, and then adjust the level of each masker so as to just mask this on-site probe; (iii) fix the masker levels at the respective MLTs obtained in stage (ii), measure the masked threshold for each probe position in turn for one masker, and do the same again for the other maskers. Repeat this three more times, reversing the testing order on each repeat. This method worked well, in the sense that the masked thresholds for the on-site probe were indeed the same for each masker. However, for the next two subjects (AB2 and AB5), this was not the case. We successfully used a modified version of the method for AB2, AB3, and AB5. The modification was as follows: (i) set one masker to its MCL and measure the masked threshold for the on-site probe; (ii) fix the probe at this masked threshold and obtain the MLT for each masker. These levels were then used in stage (iii) which measures the masked threshold for the on-site probe for all three masker types. Four adaptive runs were obtained in each case, before proceeding to measure masked thresholds for every other probe electrode, starting with electrodes closest to the masker, and completing all four measurements for each electrode before moving on to a new electrode. This new method had the advantage that we could check that the on-site masking was the same for all maskers before potentially wasting time testing other electrodes and that the subject could “home in” on each probe electrode for a fairly long time before having to switch electrodes. A minor disadvantage is that a comparison of masked thresholds between electrodes could potentially be influenced by practice or fatigue effects. However, our purpose was to compare maskers (and, specifically, the interaction between masker type and probe electrode) rather than to measure a main effect of probe electrode.

All masked thresholds and MLTs described above were estimated using a two-up one-down (MLT) or one-up two-down (masked threshold) procedure (Levitt 1971), with a step size of 1 and 0.25 dB for the first two and last four turnpoints, with the last four of a total of six turnpoints averaged to represent threshold. Thresholds and MLTs were measured and averaged over at least four runs and are reported in dB re 1 μA. Probe positions are expressed as the central electrode of the probe relative to that of the masker, with negative numbers indicating that the probe was more apical than the masker and positive numbers indicating that the probe was more basal.

### Results

#### Masker Levels for Equal Masking

The masker levels used to measure the forward-masked excitation patterns are shown in Table 3. Recall that these maskers were intended to (and, as described below, indeed did) produce equal amounts of on-site masking. These levels were significantly lower for TP_PSA than for TP_PSC, as revealed by a significant main effect of masker type (*F*(2,8) = 34.9, *p* = 0.004) and significant pairwise comparisons (TP_PSA vs TP_PSC, *p* = 0.014; TP_PSA vs MP_SYM, *p* = 0.012; TP_PSC vs MP_SYM, *p* < 0.012; all Bonferonni-corrected). This is consistent with the idea that the on-site masking was dominated by the waveform presented to the central electrode of each tripolar masker and with previous evidence that this waveform produces more excitation when its short high-amplitude portion is anodic than when it is cathodic (Macherey et al. 2008; Macherey et al. 2010).

**TABLE 3:** The levels of the maskers used in the main part of experiment 2

	Masker level, dB re 1 μA		
Subject	TP_PSA	TP_PSC	MP_SYM
AB1,e8	53.16	55.82	43.17
AB2,e4	51.60	54.93	40.75
AB3,e8	53.94	56.65	42.54
AB4,e4	46.89	47.82	43.23
AB5,e8	52.95	54.95	46.02
**Mean**	**51.71**	**54.03**	**43.14**

#### Masked Excitation Patterns

**FIG. 4:**
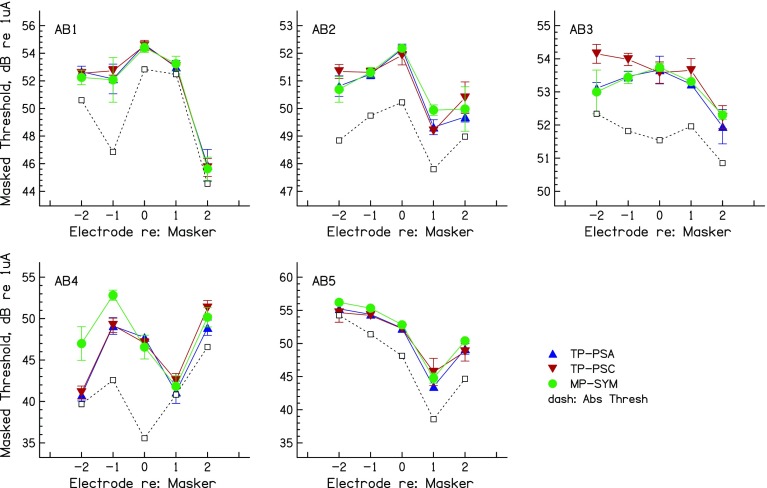
Masked excitation patterns for each subject of experiment 2, for TP_PSA (*upward triangles*), TP_PSC (*downward triangles*), and MP_SYM (*circles*) maskers. Thresholds in quiet are shown by *open squares* connected by *dashed lines*. *Error bars* are plus and minus one standard deviation, in dB.

Each panel of Figure 4 shows the masked thresholds as a function of electrode position for each masker type and for one subject. It can be seen that, as intended, masked threshold for the on-site probes were very similar for the different masker types. It is also apparent that the peak of the masked excitation pattern is not always at the on-site electrode. This could be due to differences either in the distance of each electrode from the modiolus, in the efficiency with which excitation changes are encoded at each place in the auditory nerve array, and/or in local neural survival close to each electrode. For subject AB1, these differences are reflected to some degree in the pattern of detection thresholds in quiet, shown by the dashed lines. These differences are not of great importance for comparing the spread of excitation produced by different maskers, which is the primary question addressed here. That question can be addressed by comparing the amount of off-site masking produced by the different maskers (Cosentino et al. 2015).

A repeated measures (masker type X probe position) ANOVA revealed no significant main effects (masker: *F*(2,8) = 2.03, *p* = 0.22; probe: *F*(4,16) = 1.61, *p* = 0.22); or interaction: *F*(8,32) = 0.72, *p* = 0.49). None of these effects became significant when the analysis was repeated without the on-site thresholds. However, as in experiment 1, it can be seen that the masked excitation patterns did differ between maskers for individual subjects. These differences were most noticeable for subjects AB3 and AB4, and, when the individual data were entered into separate two-way (masker type X probe position) univariate ANOVAs, using the individual runs as error terms, all subjects except AB1 showed significant main effects of masker and/or a masker-probe interaction (Table 4a). Inspection of Figure 4 suggests that these effects arose because either the TP_PSC or the MP_SYM masker produced more off-site masking than the other two maskers. Put another way, the TP_PSA masker never produced a broader spread than the other two maskers, and sometimes produced a narrower spread than at least one other masker, but there was no consistent effect across subjects. To evaluate this further, we repeated the univariate ANOVAs so as to separately compare TP_PSA with each of the other two maskers. As shown in Table 4b, c, all listeners except AB1 showed a significant (uncorrected) difference between TP_PSA and at least one other masker; as noted above and shown in Figure 4, this was always in the direction of the TP_PSA masker producing the narrower spread of excitation.

**TABLE 4:** a) Univariate ANOVA on masked excitation patterns in experiment 2 (individual subject data). b) Univariate ANOVAs (individual subjects) TP_PSA vs TP_PSC. c) Univariate ANOVAs (individual subjects) TP_PSA vs MP_SYM

Subject	Electrode effect	Masker effect	Interaction
a) All maskers			
AB 1	*F*(4,45) = 275.1, *p* < 0.001	*F*(2,45) = 0.5, n.s.	*F*(8,45) = 0.4, n.s.
AB 2	*F*(4,45) = 105.6, *p* < 0.001	*F*(2,45) = 1.8, n.s.	*F*(8,45) = 3.1, *p* < 0.01
AB 3	*F*(4,35) = 47.6, *p* < 0.001	*F*(2,45) = 13.7, *p* < 0.001	*F*(8,45) = 2.6, *p* < 0.05
AB 4	*F*(4,45) = 162.4, *p* < 0.001	*F*(2,45) = 22.2, *p* < 0.001	*F*(8,45) = 1.2, *p* < 0.001
AB 5	*F*(4,42) = 409.2, *p* < 0.001	*F*(2,42) = 8.6, *p* = 0.001	*F*(8,42) = 2.1, *p* = 0.05
b) TP_PSA vs TP_PSC			
AB 1	*F*(4,30) = 256.7, *p* < 0.001	*F*(1,30) = 0.2, n.s.	*F*(4,30) = 0.6, n.s.
AB 2	*F*(4,30) = 110.9, *p* < 0.001	*F*(1,30) = 4.0, *p* = 0.05	*F*(4,30) = 3.8, *p* < 0.05
AB 3	*F*(4,30) = 40.0, *p* < 0.001	*F*(1,30) = 22.8, *p* < 0.001	*F*(4,30) = 2.6, n.s
AB 4	*F*(4,30) = 192.0, *p* < 0.001	*F*(1,30) = 7.1, *p* < 0.05	*F*(4,30) = 3.9, *p* < 0.05
AB 5	*F*(4,28) = 201.6, *p* < 0.001	*F*(1,28) = 0.94, n.s.	*F*(4,28) = 2.6, n.s.
c) TP_PSA vs MP_SYM			
AB 1	*F*(4,30) = 134.3, *p* < 0.001	*F*(1,30) = 0.0, n.s.	*F*(4,30) = 0.2, n.s.
AB 2	*F*(4,30) = 69.9, *p* < 0.001	*F*(1,30) = 2.3, n.s.	*F*(4,30) = 1.3, n.s.
AB 3	*F*(4,30) = 27.8, *p* < 0.001	*F*(1,30) = 0.2, n.s.	*F*(1,30) = 0.7, n.s
AB 4	*F*(4,30) = 86.1, *p* < 0.001	*F*(1,30) = 29.7, *p* < 0.001	*F*(4,30) = 11.5, *p* < 0.001
AB 5	*F*(4,28) = 729.5, *p* < 0.001	*F*(1,28) = 38.9, *p* < 0.001	*F*(4,28) = 0.8, n.s.

#### Loudness as a Correlate of Spread of Excitation

**FIG. 5:**
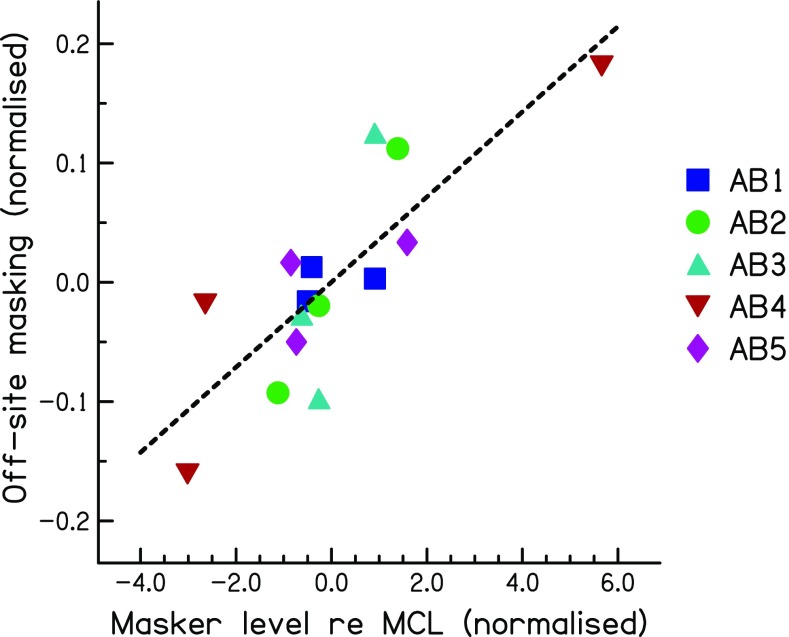
Scatter plot showing the correlation between the masker level relative to its MCL (a measure of loudness) and the amount of off-site masking (a measure of the spread of excitation). Both measures are normalised so that the mean for each listener, across masker types, is zero.

One practical limitation for measuring spread-of-excitation using masked excitation patterns is that the measurement of thresholds on multiple electrodes is time consuming. In addition, it is not possible to obtain a complete measure of spread of excitation for maskers that are at or near the edge of the electrode array, because there are insufficient probe electrodes on one side of the masker. A possible solution may come from comparing the loudness of different maskers that each produces the same amount of on-site masking. A reasonable prediction is that those maskers that produce broader excitation patterns will sound louder, because they excite more off-site neurons. To test this hypothesis, we derived measures of loudness and of spread-of-excitation for each masker and subject in experiment 1. As described in the “Method” section for experiment 2, we had already obtained the threshold and MCL for each masker as a preliminary to the main experiment. We used the actual level of the masker used in the main experiment (Table 3), in dB relative to its MCL, as a measure of the loudness of that masker. We used the average masked threshold for the off-site probes, minus that for the on-site probe, as a measure of the spread of excitation. Both of these measures were then normalised so as to remove across-subject differences, so that, for each subject, the mean of each value across masker types was equal to zero. The normalised measure of off-site minus on-site masking was further divided by the maximum amount of masking obtained (in dB), with any masker type and probe electrode, for each listener. This was done to avoid the analysis being dominated by the results of subject AB4, who showed large differences between masker types. Its effect was to reduce the correlation, which was nevertheless significant (*r* = 0.82, df = 8, *p* < 0.01) and which is shown in Figure 5. The correlation also remains significant if one instead, or additionally, removes subject AB4’s data (*t* = 0.74 and 0.71, respectively; in both cases, df = 6, *p* < 0.05) or if one expresses each masker level as a percentage of its dynamic range in dB (*r* = 0.88, df = 8, *p* < 0.01). This demonstrates that, in principle, one may be able to compare the spread of excitation produced by different maskers by comparing their loudness, provided that they produce the same masking of an on-site probe. We believe that this conclusion is likely to generalise to comparisons of maskers other than those used here, provided that the excitation patterns are expected to differ primarily in width rather than position. Exceptions might occur when comparing maskers that are expected to produce different loci of excitation, such as those generated in monopolar vs “phantom electrode” modes (Macherey and Carlyon 2012; Saoji et al. 2013), or, indeed, when the maximum of excitation shifts due to the use of asymmetric pulses in bipolar mode (experiment 1). However, in the vast majority of cases, excitation patterns peak at the same place for monopolar and tripolar stimulation, with only minor exceptions (Landsberger et al. 2012; Fielden et al. 2013; Padilla and Landsberger 2016). Experiment 3 uses the above reasoning to test for an overall difference in the spread of excitation produced by the three masker types, using a slightly different method and a larger number of masker electrodes than in experiment 1.

## **EXPERIMENT 3: ON-SITE MASKING BY EQUALLY LOUD MASKERS**

### Rationale and Method

Our analysis of experiment 2, described above, showed a correlation between spread of excitation and the loudness of maskers that produced equal on-site masking; the correlation was significant whether loudness was estimated from sound level in dB *re* MCL or as a percentage of dynamic range. A corollary of this finding, and of the argument presented in that section, is that, when two maskers are equally loud, the one that produces the narrower excitation pattern should produce the most on-site masking. This prediction holds if the on-site probe threshold is determined by the amount of masker excitation at that site: for equally loud maskers, the one that produces the narrowest excitation pattern will produce relatively more on-site excitation (and hence more on-site masking), and less off-site excitation. We therefore measured on-site masking for equally loud TP_PSA, TP_PSC, and MP_SYM maskers for the five subject/masker electrode combinations of experiment 2, plus a total of five other electrodes. The aim was to use this fast method of comparing excitation pattern widths to test a larger number of subject/masker electrode combinations and to determine whether there was a consistent overall difference between the three types of stimulation.

The subject/masker electrode combinations tested are shown in the first column of Table 5. The rows shown in bold italics are for the combinations tested in experiment 2. Two methods of equating masker loudness were used. In the majority of cases, we simply adjusted the level of each masker to its MCL, as in the preliminary stages of experiment 2. In three cases (AB3,e8, AB5,e4, and AB5,e8), we had already obtained on-site masked thresholds for the three maskers set to a slightly softer level (point 5 on the loudness chart) and those data were used here. For subject AB2, the loudness of each stimulus was formally balanced to each of the other two. The procedure used was based on the one described by Landsberger and McKay (2005). Initially, the level of the TP_PSA stimulus was set to its MCL, and the subject was instructed (in separate trials) to balance each of the other two stimuli to it by increasing and decreasing the level until confident that the test stimulus was alternately louder and softer than the reference stimulus. He then pressed a button when satisfied that the two sounds were of equal loudness. This was repeated four times, each with a slightly different initial “soft” level of the test stimulus. The entire procedure was then repeated with the TP_PSA switched to the reference stimulus and TP_PSC or MP_SYM as the fixed test stimulus. The matched loudness of the test stimulus was then taken as the level of TP_PSA plus the mean level difference obtained from all eight matches.

**TABLE 5:** The levels of the maskers obtained in the first stage of experiment 3 and used to obtain the masked thresholds shown in Figure 6. The rows shown in bold italics are for the combinations of subject and electrode tested in experiment 2

	Masker level, dB re 1 μA					
	Masker at MCL			Loudness balanced masker		
	TP_PSA	TP_PSC	MP_SYM	TP_PSA	TP_PSC	MP_SYM
AB1,e4	54.65	56.90	43.23	x	x	x
AB1,e8	***54.65***	***57.62***	***43.23***	x	x	x
AB2,e4	***53.98***	***54.81***	***42.28***	***54.17***	***55.71***	***42.48***
AB2,e10	x	x	x	55.39	57.17	44.35
AB3,e4	54.65	55.85	42.92	x	x	x
AB3,e8	***53.62***	***54.65***	***42.28***	x	x	x
AB4,e2	53.44	55.71	42.92	x	x	x
AB4,e4	***52.87***	***54.96***	***42.28***	x	x	x
AB4,e6	55.85	57.38	44.35	x	x	x
AB5,e8	***54.15***	***56.12***	***44.86***	x	x	x

Masked thresholds were obtained for each masker type, using the same method as in experiment 2. The probe was presented on the same electrode as the masker, and the final estimate for each electrode was obtained from the average of four adaptive runs. For four subjects, including a new subject, AB6, we additionally compared on-site masking for TP_PSA, TP_PSC, and a TP_SYM masker. The TP_SYM masker consisted of a 97-μs anodic phase immediately followed by a 97-μs cathodic phase. For these extra measures, the maskers were loudness-balanced using the procedure described above. For two subjects, on-site masking for loudness-balanced TP_PSA and TP_PSC maskers was additionally measured at masker-probe gaps of 5, 10, 20, 40, and 80 ms, in order to compare the decay of masker excitation for stimuli of different polarity.

### Results

**FIG. 6:**
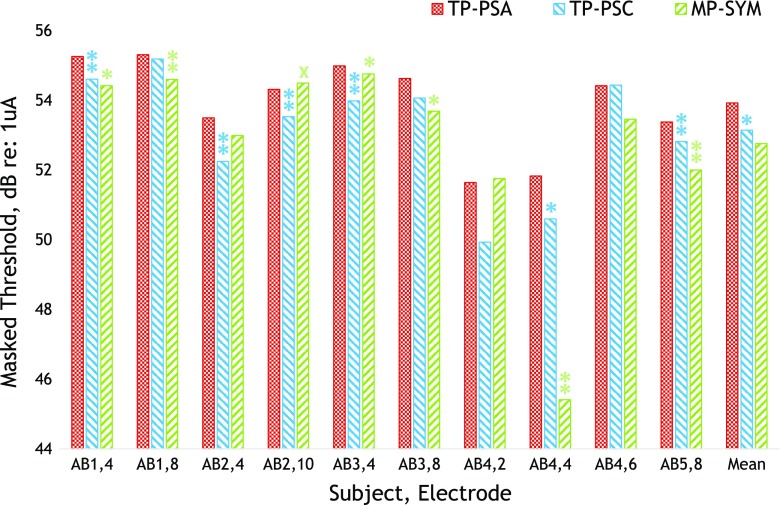
Masked thresholds for each masker type and subject/electrode combination of experiment 3. Each cluster of *three bars* shows the data for one subject/electrode combination, with conditions TP-PSA, TP_PSC, and MP_SYM ordered from *left* to *right* and shown in *red* (cross-hatch), *blue* (*downward stripes*), and *green* (upward stripes), respectively. Cases where the TP_PSC stimulus produced significantly more masking than either the TP_PSC or MP_SYM masker are shown by *single* (*p* < 0.05) or *double* (*p* < 0.01) *asterisks*, respectively. The one case where it produced significantly less masking than the MP_SYM stimulus (subject AB2, e10, *p* < 0.05) is shown by a *cross*.

The masker levels used are shown in Table 5. We obtained masked thresholds for subject AB4, electrode 4, using both methods for matching the loudness of the different maskers, and in this case, we averaged the masked thresholds from the two methods. The results are shown in Figure 6. A repeated-measures ANOVA found no main effect of masker type (*F*(2,18) = 2.61, *p* = 0.134), but Bonferonni-corrected pairwise comparisons showed that the TP_PSA masker produced significantly more masking than TP_PSC (*p* = 0.004). No other pairwise comparisons were statistically significant (TP_PSA vs MP_SYM, *p* = 0.29; TP_PSC vs MP_SYM, *p* = 1.0). We also analysed the individual subjects’ data by performing *t* tests between the TP_PSA and each of the TP_PSA and MP_SYM maskers. Instances where the TP_PSA produced significantly higher masked thresholds are shown by the asterisks in Figure 6. It can be seen that this was the case at either the 5 % (single asterisk) or the 1 % (double asterisks) level of significance in 12 out of the 20 comparisons. These comparisons are uncorrected, and one would expect one of them to be significant at the 5 % level by chance. Five of them would have remained significant after Bonferroni correction for multiple comparisons; these were the TP_PSA vs TP_PSC comparisons for subjects/electrodes AB1/e4, AB2/e10, AB3/e4, and AB5/e8 and the TP_PSA vs MP_SYM comparison for AB4/e4. In one case, subject AB2 on electrode 10, the TP_PSA stimulus produced slightly but significantly less masking than the MP_SYM masker, as shown by the single cross (*p* < 0.05).

The significant difference between TP_PSA and TP_PSC maskers is consistent with our hypothesis that the former should produce a narrower spread of excitation than the latter, due to decreased excitation at the side lobes compared to the central lobe (Fig. 2). Overall, the differences between the three maskers were generally small. An exception was subject AB4 on electrode 4, for whom the masked thresholds obtained with the MP_SYM masker were 6.4 and 5.2 dB lower than with the TP_PSA and MP_SYM maskers, respectively. This is consistent with the markedly broader masked excitation patterns for the MP_SYM masker for this subject in experiment 2 (Fig. 4). Assuming that on-site masking is a good measure of spread of excitation, then, as in experiment 2, the results show that the TP_PSA masker never produces a broader spread (lower masked threshold) than the other maskers, even though its spread is not always narrower. That is, the TP_PSA masker is a “safe bet”, but does not produce a large and consistent benefit over the MP_SYM pulse trains that are implemented clinically. It was also not significantly different from the TP_SYM masker in the four subjects for whom this was tested (Table 6). A repeated-measures ANOVA performed on the results of that experiment revealed a significant main effect of masker type (*F*(2,6) = 14.3, *p* < 0.05). Bonferonni-corrected pairwise comparisons revealed that the TP_PSA masker produced higher masked thresholds than the TP_PSC masker (*p* < 0.02) but not the TP_SYM masker (*p* = 0.81).

**TABLE 6:** Columns 2–4 show masked thresholds (dB re 1 μA) for on-site probes, for the four subjects of experiment 3 who were tested with the TP_SYM masker. The first column shows the subject and masker electrode tested. Columns 5–7 show the results of unpaired *t* tests between the two conditions shown at the top of each column. These *t* tests were performed using the four runs tested with each masker and are not corrected for multiple comparisons. Significant differences at the 5 and 1 % levels are shown by single and double asterisks, respectively

				TP_PSA	TP_PSA	TP_SYM
	TP_PSA	TP_PSC	TP_SYM	TP_PSC	TP_SYM	TP_PSC
AB1,e4	55.2	54.6	55.0			
AB2, e4	53.4	52.6	53.5	*		**
AB3,e4	55.1	54.6	54.6	*		
AB6,e6	53.8	52.9	53.7	**		*
**Mean**	**54.4**	**53.6**	**54.2**			

**FIG. 7:**
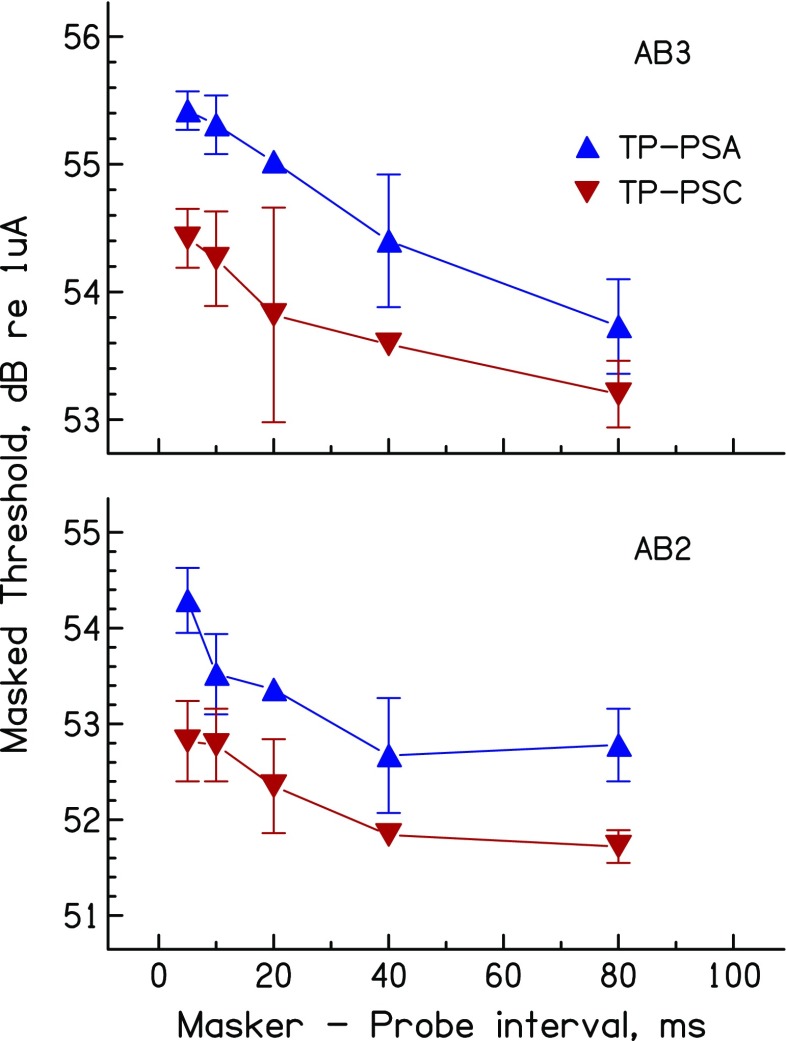
Decay of forward masking for TP_PSA and TP_PSC maskers for the two subjects tested on this measure in experiment 3.

Three assumptions underlie the use of the present method as an estimate of the spread of excitation. One of these is that the method of equating loudness for the different maskers is sufficiently accurate. Although MCL adjustments are almost certainly subject to some error, it seems unlikely that this could explain the significant difference observed in on-site masking by TP_PSA and TP_PSC maskers. That would require a systematic difference in loudness that was not driven by a difference in excitation pattern, and the most obvious alternative, a difference in the temporal response, seems unlikely given CI users’ insensitivity to timing differences at the high rates (1031 pps) used here (Shannon 1983; Townshend et al. 1987; Kong et al. 2009; Ihlefeld et al. 2015). The use of MCLs has the advantage of more closely approximating the clinical situation where fast estimates of loudness are required. Second, as noted above, the method assumes that the maskers to be compared do not differ markedly in the place of maximum excitation. A third assumption, as mentioned in the “Overview and Rationale” section above, is that the forward masked threshold of an on-site probe is determined by the amount of masker excitation at that place. If the decay of excitation differed systematically between two maskers, this could produce different amounts of forward masking even if the excitation patterns were identical. There is in fact some evidence that the decay of excitation can differ between anodic and cathodic stimuli, at least in the cat (Matsuoka et al. 2000). We therefore measured forward masking as a function of masker-probe gap for loudness-balanced TP_PSA and TP_PSC maskers, for subjects AB2 and AB3 on electrode 4. The results, shown in Figure 7, reveal no marked difference in the decay of forward masking between the two masker types.

Finally, we applied our method to the results of a study reported by Fielden et al. (2013), who generously provided us with some of their raw data. They measured forward masking produced by pairs of equally loud maskers that were centred on the probe electrode and that were presented in MP or pTP mode. These measures were obtained with the maskers loudness-balanced both at a “loud” and a “soft” level. They defined the width of the masking function as that separation at which the amount of masking was 75 % of that obtained at the maximum. Measured this way, the function width was slightly but significantly narrower for the pTP maskers, but there was no effect of overall loudness or interaction between them. Importantly for our purposes, they included a condition in which the two “maskers” were in fact interleaved on a single electrode which was the same as the probe electrode. Because the pTP and MP maskers were presented at equal loudness, we could use the difference between the on-site masked thresholds as a measure of the difference in spread of excitation. Masked thresholds from the eight subjects whose data they analysed were entered into a two-way (masker mode X loudness) repeated-measures ANOVA. The results were similar to those they reported from the complete masking functions: there was a significant effect of mode (*F*(1,7) = 15.8, *p* = 0.005) as well as, unsurprisingly, a main effect of the overall level (*F*(1,7) = 24.8, *p* = 0.001) but no interaction (*F*(1,7) = −0.03, *p* = 0.86). Hence, both methods provided evidence for greater selectivity of masking in pTP compared to MP mode, but no evidence that this effect depends on overall level.

## **DISCUSSION**

### Bimodal Excitation Profiles Elicited by Bipolar Maskers

As argued in the “INTRODUCTION”, bipolar stimulation has the potential to elicit bimodal excitation patterns, with a peak of excitation near each stimulating electrode. Experiment 1 showed that, for some subjects, there was evidence for a bimodal spread of excitation for maskers presented in bipolar mode, with only a single unused electrode between the members of each bipolar electrode pair. This bimodality was only slightly reduced by our manipulation of stimulus polarity, perhaps because polarity effects are small at the low excitation levels needed to mask the low-level probes used here and in other PTC measurements. As noted in the “INTRODUCTION”, at high excitation levels, where polarity effects are most marked, the excitations elicited by each electrode of a bipolar pair may blur together. Nevertheless, even though the excitation pattern may not be bimodal, it will be quite broad as it encompasses both stimulating electrodes.

### Effect of Polarity on Spread of Excitation with Tripolar Maskers

Experiment 3 showed that a TP_PSA masker produced significantly more on-site masking than an equally loud TP_PSC masker. As argued in the “INTRODUCTION” and illustrated in Figure 2b, c, this is consistent with our hypothesis that one can exploit CI users’ polarity sensitivity in order to manipulate the relative effectiveness of the central vs side lobes of a TP excitation pattern. Similar to the findings obtained with the BP maskers of experiment 1, the differences between results obtained with different masker types were usually small, and differed somewhat across subjects. Furthermore, for the four subjects tested in experiment 3 with TP_SYM maskers, we failed to observe a significant difference between TP_SYM and TP_PSA. This is perhaps not surprising, given our expectation that, in terms of excitation pattern spread, one might expect TP_SYM to be intermediate between TP_PSA and TP_PSC, and because the differences between these latter two stimuli were already small.

### Spread of Excitation with Multipolar vs Monopolar Maskers

There is mixed evidence for sharper excitation patterns with multi-polar (BP, TP, QPVC, AP) maskers compared to MP stimulation. In an experiment using pTP maskers, Bierer and Faulkner (2010) found that PTCs were sharper with pTP than with MP probes. In addition, Fielden et al. (2013) measured threshold for a pTP probe as a function of the separation between two equally loud maskers centred on the probe electrode and found evidence that place specificity for pTP maskers was slightly greater than that for the MP maskers. Srinivasan et al. (2010) measured masked excitation patterns using a QPVC probe, where the current was steered to positions ranging between the two central electrodes of either a QPVC masker or between a monopolar pair of electrodes (“monopolar virtual channel”). They found that excitation patterns were sharper with the QPVC masker, but it should be noted that they measured masking only between the two central electrodes, and not near the flanking electrodes; as argued above, neural excitation arising from flanking electrodes might broaden the excitation pattern. It is possible that, if Srinivasan et al. (2010) had tested a wider range of probe electrode positions, the masked excitation patterns would have had similar or even broader widths for QPVC compared to MP maskers. More recently, Padilla and Landsberger (2016) measured three-point forward-masked excitation patterns for five masker electrodes in eight subjects, for maskers in MP and pTP mode. They found that the excitation patterns were narrower in pTP mode for the vast majority of instances, although the differences were on average small and varied markedly across subjects and electrodes.

Some studies have found no evidence at all for greater spatial selectivity with multi-polar stimulation. Fielden et al. (2014) required listeners to detect differences in timing between pulse trains presented to pairs of electrodes. They measured place specificity in terms of the function relating performance to electrode separation and found no difference between the two stimulation modes. A similar negative finding was obtained by Marozeau et al. (2015) when comparing AP and MP modes. It is not clear why the conclusions have differed somewhat across studies. One possible explanation is suggested by Litvak’s (Litvak et al. 2007) model described in our “INTRODUCTION”; in TP mode, the broadening of excitation patterns produced by the side lobes is likely to be greatest when neurons responding to the central lobe are saturated. Bierer and Faulkner (2010) measured PTCs, where both the probe level and the masker excitation at the probe place are low, and where firing-rate saturation is less likely, and they did indeed see markedly sharper patterns for TP than for MP probes. The other studies described above used paradigms in which the probe threshold was higher and generally observed small or null effects. However, differences in level cannot provide a complete explanation for the differences between studies because, as described in the “Results” section of experiment 3, Fielden et al. (2013) did not find an interaction between masker mode and the width of their forward masking functions.

### Clinical and Practical Implications

It is clear from the present results that asymmetric waveforms do not provide a panacea for the failure of multi-polar stimulation to provide a consistently narrower spread of excitation than can be obtained in the monopolar mode used clinically. There were, however, individual cases where the spread of excitation could be restricted by using tripolar stimulation and also where stimulus polarity had a significant effect on the spread of excitation in TP mode. We believe the greatest practical application of the present study lies in the validation of a fast method of comparing spread of excitation that has the potential to be used across the entire electrode array. There may be cases, for example where a patient’s speech perception is poor, where it would be useful to search for electrodes—such as electrode 4 in subject AB5—where the mode of stimulation has a marked effect on the spread of excitation. In such cases, the use of a simple one-point measure of loudness may provide an efficient method of identifying electrodes where a modification to the mode of stimulation and/or waveform shape could improve spatial selectivity.
